# The Effect of Surgical Treatments for Trapeziometacarpal Osteoarthritis on Wrist Biomechanics: A Cadaver Study

**DOI:** 10.1016/j.jhsa.2019.10.003

**Published:** 2020-05

**Authors:** Darshan S. Shah, Claire Middleton, Sabahat Gurdezi, Maxim D. Horwitz, Angela E. Kedgley

**Affiliations:** ∗ Department of Bioengineering, Imperial College London, London, United Kingdom; † Department of Hand Surgery, Chelsea and Westminster Hospital, London, United Kingdom

**Keywords:** Arthroplasty, LRTI, simulator, trapeziectomy, trapeziometacarpal osteoarthritis

## Abstract

**Purpose:**

Studies have shown the effects of surgical treatments for trapeziometacarpal osteoarthritis on thumb biomechanics; however, the biomechanical effects on the wrist have not been reported. This study aimed to quantify alterations in wrist muscle forces following trapeziectomy with or without ligament reconstruction and replacement.

**Methods:**

A validated physiological wrist simulator replicated cyclic wrist motions in cadaveric specimens by applying tensile loads to 6 muscles. Muscle forces required to move the intact wrist were compared with those required after performing trapeziectomy, suture suspension arthroplasty, prosthetic replacement, and ligament reconstruction with tendon interposition (LRTI).

**Results:**

Trapeziectomy required higher abductor pollicis longus forces in flexion and higher flexor carpi radialis forces coupled with lower extensor carpi ulnaris forces in radial deviation. Of the 3 surgical reconstructions tested post-trapeziectomy, wrist muscle forces following LRTI were closest to those observed in the intact case throughout the range of all simulated motions.

**Conclusions:**

This study shows that wrist biomechanics were significantly altered following trapeziectomy, and of the reconstructions tested, LRTI most closely resembled the intact biomechanics in this cadaveric model.

**Clinical relevance:**

Trapeziectomy, as a standalone procedure in the treatment of trapeziometacarpal osteoarthritis, may result in the formation of a potentially unfilled trapezial gap, leading to higher wrist muscle forces. This biomechanical alteration could be associated with clinically important outcomes, such as pain and/or joint instability.

The trapeziometacarpal joint is the most common site requiring surgery for symptomatic osteoarthritis in the upper limb.^1^ Among the surgical methods proposed for the treatment of severe basal thumb osteoarthritis—including trapeziectomy with or without ligament reconstruction with tendon interposition (LRTI), implant arthroplasty, arthrodesis, arthroscopic resection, and metacarpal extension osteotomy—trapeziectomy, first reported in 1949,^2^ still remains a common component of many modern surgical reconstructions.^3^ However, the trapezial gap, created by resection of the trapezium, can cause persistent thumb weakness and instability, owing to potential proximal migration of the thumb.^4^ In addition, the pseudarthrosis of the first metacarpal with the scaphoid can dislocate or degenerate, leading to pain.^5^ Therefore, numerous surgical techniques have evolved to prevent the proximal displacement of the first metacarpal post-trapeziectomy, such as temporary stabilization with Kirschner wires (K-wires), prosthetic replacements, and LRTI.

Some of the oldest surgical reconstructions include the use of silicone elastomer implants.^6^ Although their designs attempt to preserve the natural anatomy and biomechanics of the joint,^7^ these implants have been known to suffer from a few limitations, such as implant loosening.^8^ Wrist tendons, such as the flexor carpi radialis (FCR), have been used as a graft to create a tendon sling for the stabilization of the first metacarpal^9^; however, tendon slings might not be adequate to prevent proximal migration of the first metacarpal if they yield under load.^4^ To combine features of a silicone implant and tendon slings, the tendon tie-in implant has been proposed post-trapeziectomy, wherein the tendon sling is wrapped around the base of the implant, with the goal of preventing dislocation.^10^

The modern surgical technique of LRTI involves the use of the FCR or other tendons for ligament reconstruction, with varying knot designs for tendon interposition.^4^^,^^11^^,^^12^ Studies have shown that LRTI using the FCR results in decreased pain and increased grip strength and key pinch strength,^11^ as well as smaller proximal displacement of the first metacarpal.^13^ Owing to the stability it provides without the incorporation of an implant, LRTI is preferred over other surgical reconstructions.^14^ However, studies have questioned the success of LRTI^15^, ^16^, ^17^ and have proposed a suture suspension arthroplasty procedure as a faster and less-invasive alternative to harvesting a tendon graft.^18^

Notwithstanding the growing popularity of LRTI to treat trapeziometacarpal osteoarthritis, each of the commonly performed surgical interventions has associated advantages and limitations. Despite the reported shortcomings of trapeziectomy,^4^^,^^5^ *in vivo* studies based on the dimensions of the trapezial gap, thumb pain, and thumb strength^13^^,^^19^ question the need of surgical reconstructions post-trapeziectomy.^20^ *In vitro* studies comparing these reconstructions by analyzing joint kinematics and biomechanics are limited by the drawback of applying passive or constant loads to the muscles during the experimental protocol.^21^^,^^22^ Therefore, the primary objective of this study was to simulate dynamic wrist motions on a physiological simulator using active loads to compare a range of surgical techniques and to quantify the effect of surgical reconstructions commonly used to treat trapeziometacarpal osteoarthritis on wrist biomechanics by comparing wrist muscle forces for each condition. We hypothesized that, owing to the alteration of the trapezial gap, surgical intervention would cause a rise in the muscle forces of the radial flexors—FCR and abductor pollicis longus (APL)—thereby altering the distribution of muscle forces in the wrist from those observed in the intact case.

## Methods

### Specimen preparation

Nine fresh-frozen cadaveric specimens—7 women and 2 men (mean age, 50.7 years; range, 31–59 years)—with no traumatic or musculoskeletal degenerative pathology, were obtained from a licensed human tissue facility. Ethical approval was obtained from the institutional tissue management committee according to the Human Tissue Act. The specimens, stored at –20°C prior to this study, were thawed at room temperature for 12 hours. The 6 wrist muscles considered for this study—FCR, flexor carpi ulnaris (FCU), extensor carpi radialis longus (ECRL), extensor carpi radialis brevis (ECRB), extensor carpi ulnaris (ECU), and APL—were identified and dissected at their distal myotendinous junction. All other soft tissue was resected 5 cm proximal to the wrist, thereby preserving the wrist capsule and the retinaculum. The elbow was fixed in 90° flexion with neutral forearm rotation (pronation angle, 0°) using K-wires, while all digits were left unconstrained.

### Experimental setup

**Figure 1:**
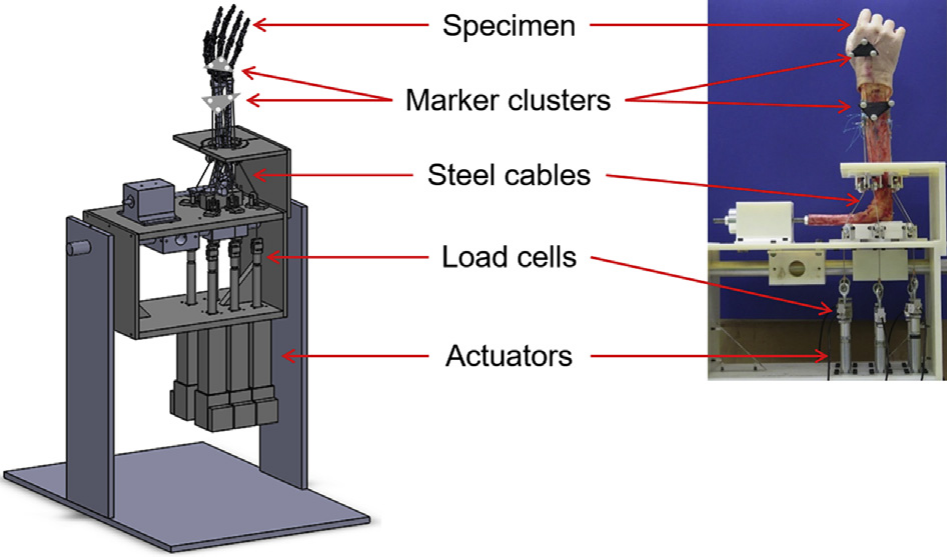
Schematic of the physiological wrist simulator.

Specimens were mounted on a physiological wrist simulator (Fig. 1).^23^ Six linear actuators (SMS Machine Automation, Barnsley, UK) mounted in-line with servo motors (Animatics Corp., Milpitas, CA) were used to re-create wrist motions by applying tensile loads to steel cables sutured to the tendons of the 6 muscles. Tendon forces were measured using load cells (Applied Measurements Ltd., Aldermaston, UK) connected in series with the actuators. Clusters of retroreflective passive markers fixed rigidly to the third metacarpal and the radius were registered using anatomical landmarks recommended by the International Society of Biomechanics^24^ to define the coordinate systems of the hand and the forearm, respectively. Joint angles were obtained in real time using an 8-camera optical motion capture system (Qualisys, Göteborg, Sweden).

Active wrist motions were simulated *in vitro* by means of hybrid control, which used position feedback to drive joint kinematics according to the input set point waveform, with simultaneous force feedback to ensure muscle forces remained within physiological bounds.^25^ The control strategy minimized kinematic error by computing the distribution of actuator displacements across the 6 muscles to achieve the desired joint kinematics every 4 to 5 ms.^25^ Specimen-specific moment arms of the muscles, determined according to the tendon excursion method prior to active simulations,^26^ were used as custom inputs. Lower bounds on muscle forces were chosen according to values for minimum muscle activity obtained from EMG,^27^ and upper bounds on muscle forces were defined as the product of muscle physiological cross-sectional area^28^ and specific muscle tension^29^ (Table 1).

**Table 1:** Bounds on Tendon Forces

Muscle	Lower Bound (N)	Physiological Cross-Sectional Area^28^ [A] (cm^2^)	Specific Muscle Tension^29^ [B] (N/cm^2^)	Upper Bound [A*B] (N)
FCR	10	3.9	25	97.5
FCU	10	6.6	25	165.0
ECRL	10	2.5	25	62.5
ECRB	10	2.7	25	67.5
ECU	10	2.3	25	57.5
APL	10	1.7	25	42.5

### *In vitro* simulations

Six cycles of each planar wrist motion, including flexion-extension (FE), 50° flexion to 30° extension to 50° flexion (FE-5030) and radioulnar deviation (RUD), 15° ulnar deviation to 15° radial deviation to 15° ulnar deviation (RUD-15), were simulated on intact specimens with the hand in the vertically upward orientation (Fig. 1).

This was followed by trapeziectomy, performed by excising the trapezium, intact or in a piecemeal fashion, using a Wagner volar-radial approach.^2^ Care was taken to avoid the disruption of the distal tendons insertions of APL and FCR on the first and second metacarpals, respectively. Following the removal of the trapezium, the joint capsule and superficial tissue were carefully sutured (Ethibond Excel 2-0; Ethicon Inc., Bridgewater, NJ), and active cyclic wrist motions were simulated on all specimens (Table 2).

**Table 2:** Peak Forces and Mean Forces of All Tendons During Cyclic Wrist Motions∗

Cases	Peak Force (N)					
	FCR	FCU	ECRL	ECRB	ECU	APL
**FE-5030**						
Intact	39.3 ± 7.5	27.1 ± 7.9	30.9 ± 10.9	57.1 ± 15.8	65.9 ± 6.0	26.5 ± 10.1
Trap.	47.7 ± 14.2	28.8 ± 8.1	33.7 ± 12.7	57.2 ± 13.2	61.3 ± 7.0	37.5 ± 19.9
SSA	44.6 ± 12.7	28.2 ± 8.2	32.4 ± 11.6	59.1 ± 12.9	62.7 ± 4.3	30.3 ± 12.8
PR	44.2 ± 8.5	29.4 ± 8.9	30.7 ± 9.5	58.9 ± 13.9	64.2 ± 5.6	**17.9 ± 4.5**
LRTI	39.1 ± 10.4	30.0 ± 10.0	33.0 ± 7.8	60.9 ± 13.2	64.9 ± 5.8	27.1 ± 13.5
**RUD-15**						
Intact	25.4 ± 8.6	29.6 ± 8.1	22.3 ± 6.4	37.7 ± 13.7	59.6 ± 10.9	30.8 ± 14.1
Trap.	**30.2 ± 10.1**	31.0 ± 8.0	23.9 ± 6.2	38.3 ± 13.4	55.1 ± 14.5	40.2 ± 20.8
SSA	25.3 ± 8.5	30.1 ± 8.3	21.6 ± 5.6	37.4 ± 10.1	58.0 ± 12.5	40.9 ± 13.0
PR	29.2 ± 10.9	31.1 ± 7.0	21.1 ± 5.1	39.7 ± 15.0	60.2 ± 13.4	**18.8 ± 6.3**
LRTI	20.6 ± 6.8	29.1 ± 5.7	21.7 ± 6.0	41.7 ± 14.5	61.0 ± 11.3	34.3 ± 17.4

Cases	Mean Force (N)					
	FCR	FCU	ECRL	ECRB	ECU	APL
**FE-5030**						
Intact	19.9 ± 3.5	16.2 ± 3.1	17.6 ± 3.8	32.6 ± 6.3	42.6 ± 5.1	14.4 ± 4.2
Trap.	24.0 ± 6.9	17.1 ± 4.2	19.1 ± 5.7	32.5 ± 6.1	42.8 ± 7.4	17.3 ± 5.3
SSA	23.2 ± 5.6	16.7 ± 3.5	18.6 ± 4.9	33.7 ± 5.9	43.6 ± 6.1	16.3 ± 3.6
PR	23.2 ± 5.5	17.0 ± 3.7	17.5 ± 4.4	33.4 ± 6.6	44.0 ± 6.3	11.7 ± 1.8
LRTI	21.2 ± 4.8	16.6 ± 4.2	18.3 ± 3.4	34.0 ± 5.7	43.4 ± 5.1	14.3 ± 3.9
**RUD-15**						
Intact	15.7 ± 3.9	16.7 ± 2.3	15.0 ± 3.6	26.8 ± 8.2	37.4 ± 5.8	15.9 ± 4.4
Trap.	**17.7 ± 4.6**	18.1 ± 3.3	15.5 ± 3.5	25.9 ± 7.6	**32.8 ± 6.7**	15.6 ± 4.4
SSA	16.0 ± 4.5	17.4 ± 3.1	14.4 ± 3.1	26.1 ± 6.5	36.2 ± 7.0	18.7 ± 3.8
PR	17.2 ± 5.3	18.1 ± 1.9	13.7 ± 2.9	27.3 ± 9.7	**35.1 ± 6.1**	**11.6 ± 2.2**
LRTI	13.7 ± 3.6	17.9 ± 2.6	14.0 ± 3.3	28.7 ± 9.5	37.0 ± 5.3	16.6 ± 4.8

PR, prosthetic replacement; SSA, suture suspension arthroplasty; Trap., trapeziectomy.

∗ Data are represented as mean ± 1 SD across specimens. Bold text indicates statistically significant differences between a surgical reconstruction and the intact case (*P* < .01).

Three types of surgical reconstructions post-trapeziectomy were sequentially performed in order to fill the gap created by the removal of the trapezium—suture suspension arthroplasty^30^ in 9 specimens, prosthetic replacement^10^ in 7 specimens, and LRTI^11^ in 6 specimens. Suture suspension arthroplasty was performed as described by DelSignore and Accardi,^30^ with the first metacarpal stabilized by suture slings (Ethibond Excel 0) between the FCR and the APL. A silicone implant (Tie-In Trapezium Implant; Wright Medical Technology Inc, Memphis, TN) was used as the prosthetic replacement, with the arthroplasty performed according to manufacturer guidelines. To stabilize the implant, the distal tendon of the FCR was split longitudinally into 2 portions to the base of the index metacarpal, with 1 portion resected at the musculotendinous junction, brought out into the gap left after resecting the trapezium, and tied around the waist of the implant, as prescribed by Avisar et al.^10^ The LRTI was performed according to the technique developed by Scheker and Boland,^11^ and employed the previously retracted portion of the distal tendon of the FCR to stabilize the first metacarpal.

Following each of the surgical reconstructions post-trapeziectomy, the joint capsule and surrounding tissues were carefully sutured (Ethibond Excel 2-0) before simulating cycles of wrist motions.

### Data analysis

Each specimen was moved through 6 cycles for all wrist motions. The first cycle was neglected to avoid any transient effects at the beginning of the motion, and the mean of the remaining 5 cycles was used for data analysis. Mean muscle forces across all specimens evaluated as a function of joint kinematics at every 10° in FE and 5° in RUD, as well as the mean and peak muscle forces over the entire range of motion for all specimens, were computed for each surgical reconstruction and compared with values obtained for intact specimens. When checked for normality using the Shapiro-Wilk test, the force data were found to deviate from the normal distribution. Therefore, nonparametric tests were used to compare the data. The Friedman test was performed to determine differences between muscle forces obtained during active motions simulated across the intact and the various surgically reconstructed conditions (*P* < .05). If significant interactions were observed in the Friedman test, a post hoc analysis was performed using the Wilcoxon signed-rank test, with a Bonferroni adjustment for multiple comparisons, to observe pairwise differences within groups (*P* < .01). Differences in muscle forces larger than 10% were considered clinically important. With differences in muscle forces between the intact state and the surgically altered state estimated at 5 N from our previous work using the same experimental protocol,^31^ the sample size estimate was that 6 specimens were sufficient to detect statistically significant differences (*P* < .05) with a power of 80%.

## Results

**Figure 2:**
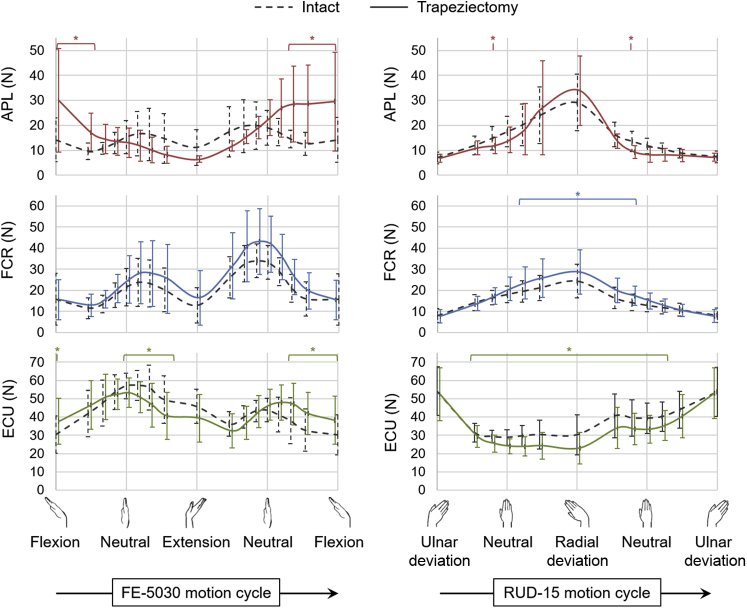
Mean muscle forces of the APL, FCR, and ECU across 9 specimens during FE-5030 and RUD-15 in the intact specimens (dashed lines) and following trapeziectomy (solid lines). Error bars represent 1 SD. The asterisk (*) represents statistically significant differences between trapeziectomy and intact cases (*P* < .01).

While simulating FE-5030 after performing trapeziectomy (Fig. 2), the APL force was higher by 112% at 50° flexion (*P* < .01) compared with that from the intact specimens. No differences were observed in mean and peak forces of the FCR, FCU, ECRL, ECRB, and ECU following trapeziectomy. In the case of RUD-15 following trapeziectomy (Fig. 2), the FCR force was higher by 18% (*P* < .01), and that of the ECU was lower by 24% (*P* < .01) at 15° radial deviation. No differences were observed in mean and peak forces of the FCU, ECRL, ECRB, and APL following trapeziectomy.

**Figure 3:**
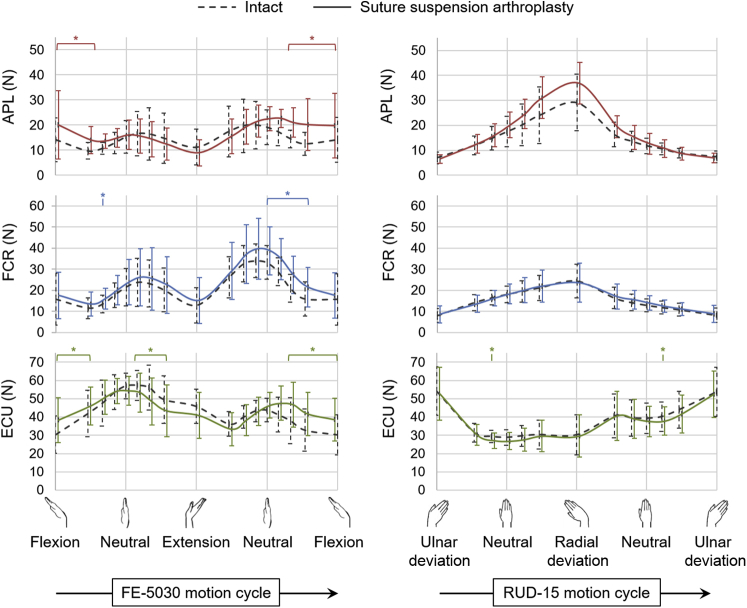
Mean muscle forces of the APL, FCR, and ECU across 9 specimens during FE-5030 and RUD-15 in the intact specimens (dashed lines) and following suture suspension arthroplasty (solid lines). Error bars represent 1 SD. The asterisk (*) represents statistically significant differences between suture suspension arthroplasty and intact cases (*P* < .01).

For FE-5030 after performing suture suspension arthroplasty post-trapeziectomy (Fig. 3), the APL force was higher by 40% at 50° flexion (*P* < .01), compared with that from the intact specimens. No differences were observed in mean and peak forces of the FCU, ECRL, ECRB, and ECU following suture suspension arthroplasty. In the case of RUD-15 (Fig. 3), no differences were observed when comparing the suture suspension arthroplasty with the intact values for mean and peak forces of any muscle throughout the range of motion.

**Figure 4:**
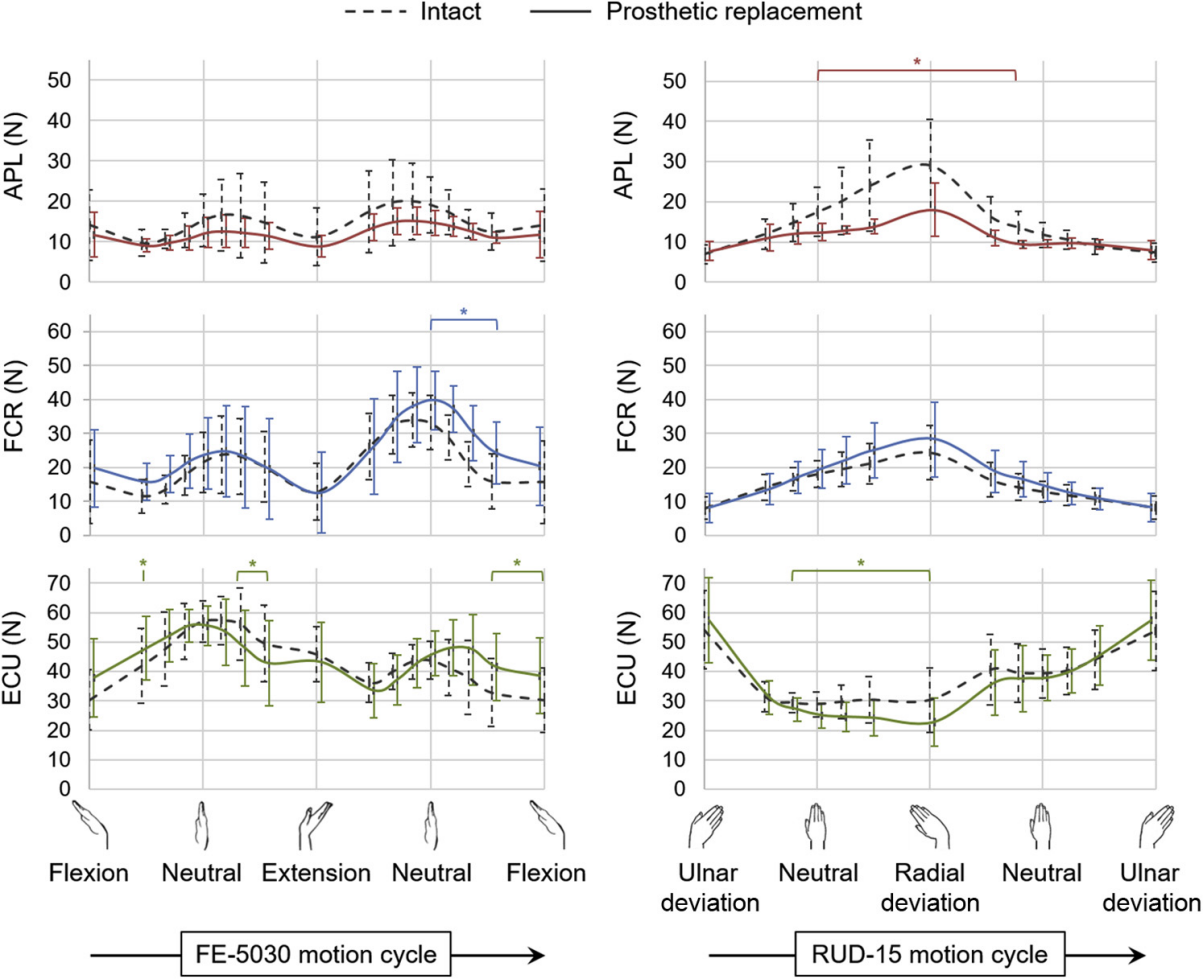
Mean muscle forces of the APL, FCR, and ECU across 7 specimens during FE-5030 and RUD-15 in the intact specimens (dashed lines) and following prosthetic replacement (solid lines). Error bars represent 1 SD. The asterisk (*) represents statistically significant differences between prosthetic replacement and intact cases (*P* < .01).

In the case of inserting the silicone implant post-trapeziectomy (Fig. 4), the peak APL force was lower by 32% (*P* < .01) during FE-5030, compared with that from the intact specimens. No differences were observed in mean and peak forces of the FCR, FCU, ECRL, ECRB, and ECU following the implant insertion. In the case of RUD-15 following implant insertion (Fig. 4), the forces of APL and ECU were lower by 33% (*P* < .05) and 21% (*P* < .01) respectively, at 15° radial deviation. No differences were observed in peak forces of the FCR, FCU, ECRL, and ECRB following implant insertion.

**Figure 5:**
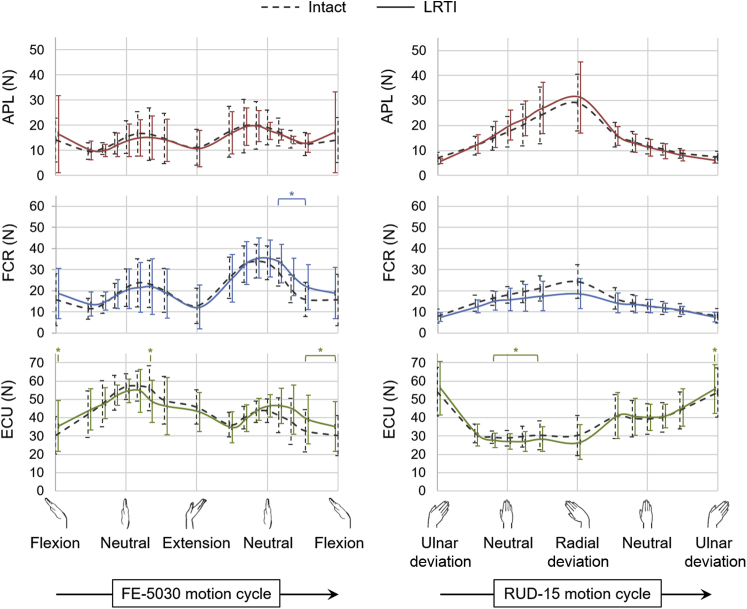
Mean muscle forces of the APL, FCR, and ECU across 6 specimens during FE-5030) and RUD-15 in the intact specimens (dashed lines) and following LRTI (solid lines). Error bars represent 1 SD. The asterisk (*) represents statistically significant differences between LRTI and intact cases (*P* < .01).

In the case of FE-5030 and RUD-15 performed following LRTI post-trapeziectomy (Fig. 5), no differences were observed for mean and peak forces of any muscle throughout the ranges of motion compared with that from the intact specimens.

## Discussion

A validated physiological wrist simulator^23^ was used to measure the alterations to wrist biomechanics caused by surgical reconstructions employed in the treatment of trapeziometacarpal osteoarthritis. Wrist motions were replicated *in vitro* using a control strategy previously shown to have low kinematic error and high repeatability.^23^^,^^25^ Results from multiple cyclic wrist motions simulated in the specimens before and after trapeziectomy showed significant changes in the wrist muscle force distribution. Owing to the absence of any external loading or nonextreme ranges of motion during the cadaveric simulations, differences in muscle forces observed between the intact condition and the postreconstruction were considered clinically important if they differed in magnitude by 10% and were statistically significant.

The removal of the trapezium resulted in a mean rise of 112% (range, 50%–210%) in APL force for high flexion angles (Fig. 2). This could be attributed to the proximal migration of the APL insertion on the base of the first metacarpal following trapeziectomy, leading to a decrease in the moment arm of the APL tendon about the FE axis of the wrist, thereby necessitating a higher force to generate the same balancing torque. The APL has the propensity to cause the greatest dorsoradial misalignment of the first metacarpal at the trapeziometacarpal joint, owing to the large resultant moment created by its point of insertion and line of action.^32^ This rise in APL force could potentially cause radial subluxation of the pseudarthrosis between the first metacarpal and the scaphoid during wrist motions involving deep flexion, which could eventually lead to the dislocation or degeneration resulting in pain, as is reported clinically*.*^5^ Moreover, significant alterations in the wrist muscle forces during certain wrist motions—for instance, higher forces of APL and ECU during flexion, or higher FCR forces coupled with lower ECU forces during radial deviation (Fig. 2)—could result in lower ranges of motion owing to pain or muscle fatigue, as well as altered carpal biomechanics.

In our *in vitro* study, the biomechanical analysis reflected the results of the surgical reconstruction immediately after it was performed, as opposed to clinical studies, which are conducted several weeks after surgery.^13^^,^^19^ However, the significant postsurgical alterations in muscle forces reflected in this study, observed especially during limited range of motion simulations without external loading, could explain clinically important outcomes over a longer period of time. Hence, these alterations should be taken into consideration when selecting treatment for younger patients.

Surgical reconstructions post-trapeziectomy were performed in the same sequence on each specimen. Suture suspension arthroplasty was selected as the first reconstruction post-trapeziectomy because it was least invasive than the 2 other reconstructions. In contrast, the stem of the silicone implant required a longitudinal hole to be drilled in the metacarpal,^10^ which was eventually used as 1 of the tunnels required for the LRTI procedure.^11^ Moreover, the portion of the distal FCR tendon retracted distally to stabilize the silicone implant^10^ was reused to fill the trapezial gap in the LRTI procedure.^11^ Thus, the effect of simulating multiple surgical reconstructions on the outcome of the experiment was expected to be minimal because the sequence of surgical reconstructions was carefully chosen such that invasive steps, such as drilling the bone, from a previous reconstruction were used for the following one. Whereas trapeziectomy and suture suspension arthroplasty were performed on all specimens, prosthetic replacement and LRTI could not be tested on 2 and 3 specimens, respectively, owing to experimental, surgical, and temporal challenges resulting from the sequential nature of the protocol.

Surgical reconstructions performed post-trapeziectomy perturbed the wrist muscle forces to varying degrees compared with the intact condition. Suture suspension arthroplasty was efficient in restoring wrist muscle forces to the intact state in RUD, but not in FE (Fig. 3). This reconstruction has been suggested as having merit based on its attributes such as being a less-invasive as well as a faster procedure than using a prosthetic replacement or LRTI^18^; however, the suture slings were probably unable to prevent the proximal migration of the first metacarpal during active wrist motions, thereby resulting in higher forces in FE.^30^ A reduction in APL force following prosthetic replacement, despite using a silicone implant of the same size for all specimens, could suggest a partial restoration of the APL insertion and tendon moment arm by filling the trapezial void; however, there was an associated increase in FCR force, especially in FE (Fig. 4). In contrast, LRTI resulted in muscle forces similar to those obtained in the intact case (Fig. 5), potentially by providing a biomechanically efficient trapezial gap restoration.

Notwithstanding their varying success in restoring joint biomechanics *in vitro*, these procedures have been proven to have certain limitations clinically.^17^ Despite LRTI being a preferred surgical reconstruction, owing to the additional suspension support provided by this technique,^14^ several clinical studies have reported no functional benefit of LRTI post-trapeziectomy.^13^^,^^15^^,^^16^^,^^19^

The *in vitro* study by Luria et al^22^ reported LRTI to be less efficient than prosthetic implants in preventing the proximal migration of the first metacarpal, while also suggesting that LRTI had no biomechanical advantage over stand-alone trapeziectomy. These outcomes contrast with the observations made in our study and may have arisen from the difference in the surgical procedures in each study—while Luria et al^22^ replicated the LRTI technique suggested by Burton and Pellegrini,^33^ a more recently proposed reconstruction by Scheker and Boland^11^ was implemented in our study, which included a sturdier tendon interposition technique specifically to prevent the proximal migration of the first metacarpal. The improved stabilization of the first metacarpal might have aided the restoration of the wrist muscle forces *in vitro*. Moreover, this reconstruction facilitated the preservation of a portion of the distal FCR tendon, thereby avoiding inherent biomechanical alterations in the joint owing to the absence of the FCR.^31^

There were limitations to this study. First, only 6 muscles inserting on the metacarpals were actuated to simulate wrist motions *in vitro*. *In vivo*, extrinsic muscles of the hand—such as flexor digitorum superficialis, flexor digitorum profundus, flexor pollicis longus, and extensor digitorum communis—would also contribute to wrist torque. In addition to the extrinsic muscles, active actuation of the intrinsic muscles of the thumb would facilitate the simulation of isolated motions of the trapeziometacarpal joint and should be included in future experiments. Second, finite cycles of planar wrist motions were simulated on an unloaded joint. Simulating multiple cycles of complex wrist motions or implementing cyclic loading might result in effects such as implant loosening or even knot loosening in the case of LRTI. Third, the analysis in this study was based on restoration of wrist muscle forces. Other biomechanical parameters, such as joint laxity, narrowing of the joint space, tendon excursions, and joint contact forces, could be quantified in future. Moreover, tracking the kinematics of individual carpal bones would enable a deeper insight into other wrist pathologies that may occur following trapeziectomy, such as dorsal intercalated segment instability.^34^

In conclusion, leaving the trapezial gap unfilled after trapeziectomy resulted in altered muscle forces during planar wrist motions, which could have implications for carpal and wrist biomechanics over time. Although clinical studies comparing LRTI with trapeziectomy suggest no clinical difference, correcting the biomechanics may improve outcomes in the younger high-demand patient. With varying success of surgical reconstructions post-trapeziectomy to stabilize the first metacarpal, further research is required to identify the ideal treatment for trapeziometacarpal osteoarthritis.
